# Inhibition of nitric oxide production of activated mice peritoneal macrophages is independent of the *Toxoplasma gondii* strain

**DOI:** 10.1590/0074-02760200417

**Published:** 2021-03-10

**Authors:** João Cláudio Damasceno-Sá, Fernanda Silva de Souza, Thiago Alves Teixeira dos Santos, Fábio Conceição de Oliveira, Maria de Fátima Sarro da Silva, Raul Ramos Furtado Dias, Wanderley de Souza, Andrea Cristina Veto Arnholdt, Sergio Henrique Seabra, Renato Augusto DaMatta

**Affiliations:** 1 Universidade Estadual do Norte Fluminense, Centro de Biociências e Biotecnologia, Laboratório de Biologia Celular e Tecidual, Campos dos Goytacazes, RJ, Brasil; 2 Centro Universitário Estadual da Zona Oeste, Colegiado de Ciências Biológicas e da Saúde, Laboratório de Tecnologia em Bioquímica e Microscopia, Rio de Janeiro, RJ, Brasil; 3 Universidade Federal do Rio de Janeiro, Instituto de Biofísica Carlos Chagas Filho, Laboratório de Ultraestrutura Celular Hertha Meyer, Rio de Janeiro, RJ, Brasil; 4 Universidade Estadual do Norte Fluminense, Centro de Biociências e Biotecnologia, Laboratório de Biologia do Reconhecer, Campos dos Goytacazes, RJ, Brasil

**Keywords:** activated macrophages, nitric oxide, inducible nitric oxide synthase, TGF-ꞵ signaling, LC3, virulence

## Abstract

**BACKGROUND:**

*Toxoplasma gondii* causes toxoplasmosis and is controlled by activated macrophages. However, infection of macrophages by tachyzoites induces TGF-β signaling (TGF-s) inhibiting nitric oxide (NO) production. NO inhibition may be a general escape mechanism of distinct *T. gondii* strains.

**OBJECTIVES:**

To evaluate in activated macrophages the capacity of *T. gondii* strains of different virulence and genetics (RH, type I; ME-49, type II; VEG, type III; P-Br, recombinant) to evade the NO microbicidal defense system and determine LC3 loading to the parasitophorous vacuole.

**METHODS:**

Activated peritoneal macrophages were infected with the different *T. gondii* strains, NO-production was evaluated by the Griess reagent, and inducible nitric oxide synthase expression, TGF-s, and LC3 localisation assayed by immunofluorescence.

**FINDINGS:**

Only RH persisted in macrophages, while VEG was more resistant than P-Br and ME-49. All strains induced TGF-s, degradation of inducible nitric oxide synthase, and NO-production inhibition from 2 to 24 h of infection, but only RH sustained these alterations for 48 h. By 24 h of infection, TGF-s lowered in macrophages infected by ME-49, and P-Br, and NO-production recovered, while VEG sustained TGF-s and NO-production inhibition longer. LC3 loading to parasitophorous vacuole was strain-dependent: higher for ME-49, P-Br and VEG, lower for RH. All strains inhibited NO-production, but only RH sustained this effect probably because it persisted in macrophages due to additional evasive mechanisms as lower LC3 loading to parasitophorous vacuole.

**MAIN CONCLUSIONS:**

These results support that *T. gondii* can escape the NO microbicidal defense system at the initial phase of the infection, but only the virulent strain sustain this evasion mechanism.

*Toxoplasma gondii* is an intracellular parasite with wide world distribution in warmblood vertebrates causing toxoplasmosis. Most infections are latent and do not cause clinical manifestations.^1^ However, the parasite can kill immune-compromised individuals and cause fetal problems.^1^ *T. gondii* strains isolated in North America and Europe are classically divided into three genetically clonal lineages: I, II, and III.^2^ Type I strains are highly virulent in mice, while type II and type III are less.^3^ ^,^ ^4^ However, most isolates from South America are biologically and genetically different from those isolated from North America and Europe.^5^ These strains are known as “recombinant”, with a few being characterised by molecular analysis and infection in mice. P-Br is a strain classified as type I-III that shows low virulence and is cystogenic in mice.^6^

*T. gondii* infects the host cell by active invasion and forms a parasitophorous vacuole (PV) that does not fuse with endolysosomal compartments, protecting the parasite from host digestion.^7^ ^,^ ^8^ The absence of compartment fusion to the PV is related to proteins secreted by specialised organelles such as rhoptries.^9^ Some of these proteins, from the serine/threonine kinase family^10^ ^,^ ^11^ ^,^ ^12^ ^,^ ^13^ are incorporated in the PV membrane providing identity and avoiding fusion with the endolysosomal compartments.^14^ Rhoptry proteins, particularly ROP5 and ROP18 of type I *T. gondii* strains.^15^ ^,^ ^16^ are virulence factors that disarm the immunity-related GTPases (IRG) microbicidal system of mouse host cells activated by interferon-gamma (IFN-γ).^12^ Strains of low virulence are destroyed in IFN-γ activated mouse host cells by the action of IRG that associate to the PV causing vesiculation and rupture of its membrane.^13^ ^,^ ^17^ ^,^ ^18^ Recruitment of IRG to the PV depends on core autophagy proteins that decorate the PV with LC3.^19^ ^,^ ^20^ ^,^ ^21^ However, this LC3 decoration is not classical autophagy, it is a cellular function known as “autophagy-related processes”.^22^ LC3 is conjugated to phosphatidylethanolamine by ubiquitin-like enzymes of the autophagy pathway, loads to the PV, and is crucial for IRG to control *T. gondii* replication.^20^ ^,^ ^21^ ^,^ ^23^ These studies used only type II *T. gondii* strains, which are avirulent to mice, to analyse the involvement of LC3 in the loading of IRG to the PV and *T. gondii* growth control. Only one study compared types I, II, and III *T. gondii* strain in a human cell system activated with IFN-γ showing that LC3, and other autophagy adaptor proteins, are loaded to the PV in a strain-specific manner, with type I strain better avoiding this decoration.^24^ Therefore, nothing is known about strain differences on the LC3 loading to the PV after infection of activated mouse peritoneal macrophages.

Macrophages activated with IFN-γ express inducible nitric oxide (NO) synthase (iNOS) that produces NO, a microbicidal agent that controls *T. gondii* growth.^25^ ^,^ ^26^ ^,^ ^27^ ^,^ ^28^ ^,^ ^29^ ^,^ ^30^ ^,^ ^31^ However, *T. gondii* infection of macrophages inhibits the production of NO^27^ ^,^ ^28^ ^,^ ^29^ ^,^ ^30^ ^,^ ^31^ ^,^ ^32^ ^,^ ^33^ ^,^ ^34^ ^,^ ^35^ by degrading iNOS expression^27^ ^,^ ^28^ ^,^ ^30^ ^,^ ^31^ ^,^ ^33^ through the proteasomal pathway.^36^ We have recently demonstrated that depending on the macrophage lineage *T. gondii* infection does not involve iNOS degradation, but NO production inhibition is constant.^33^ Also, ROPs (5, 16, 17, 17-18, 18) were not essential for NO production inhibition.^33^ TGF-β signaling is involved in the inhibition of NO production.^31^ NO is a microbicidal agent crucial in vertebrates including mammals and birds that are hosts for *T. gondii*. Therefore, most *T. gondii* strains may need to deal with this microbicidal system. However, nothing is known about the induction of TGF-β signaling and avoidance of NO production of activated macrophages by distinct *T. gondii* strains. Hence, we investigated the persistence, NO inhibition, iNOS expression, TGF-β signaling, and LC3 loading to the PV, using four strains of *T. gondii* of different genetic backgrounds (RH, ME-49, VEG, P-Br) after infection of activated mice peritoneal macrophages to determine if NO inhibition is a possible general immune evasion strategy for this parasite.

## MATERIALS AND METHODS

*Macrophages and activation* - Macrophages from the peritoneal cavity of male Swiss mice (6 weeks) were seeded on glass coverslips in 24-well plates. After 1 h at 37ºC in a 5% CO_2_ atmosphere, cells were washed and cultured for 24 h in Dulbecco’s modified Eagle’s medium (DMEM) containing 10% fetal bovine serum, 100 U/mL recombinant mouse IFN-γ (Sigma) and 0.1 µg/mL of *Escherichia coli* lipopolysaccharide (0111:B4, LPS, Sigma) to activate macrophages.^30^

*Ethical statement* - This study was carried out in strict accordance with the animal experimentation Brazilian Law #11794/08. The protocol was reviewed and approved by the Committee on the Ethics of Animal Experiments of the Universidade Estadual do Norte Fluminense (UENF) (Permit Number: 98). Mice were euthanised with CO_2_ by following the recommendations in the Guide for the Care and Use of Laboratory Animals of the National Institutes of Health (NIH).

*Parasites and animals* - Tachyzoites of the RH, ME-49, VEG, and P-Br strains were maintained by serial passage in Vero cells. Highly infected Vero cells without free tachyzoites in the supernatant were washed twice, scraped with a rubber policeman, and mechanically ruptured by passage through a syringe with a 26G needle, and the supernatant was filtered (3 µm - Millipore membrane). The supernatant, containing tachyzoites, was collected and further centrifuged at 1000g for 10 min at 4ºC. Parasites were resuspended in DMEM.

*In vitro interactions and quantification of parasitic infection and persistence* - Activated macrophages were washed, and infected with a 10:1 *T. gondii*:macrophage ratio. After 2 h, cells were washed and DMEM supplemented with foetal bovine serum (FBS), IFN-γ, and LPS were added. The cells were further cultivated for 24 and 48 h. For quantification of the infection (2 h) and parasitic persistence (24 and 48 h) macrophages were fixed in phosphate-buffered saline (PBS) containing 3% formaldehyde, washed and stained with a 10% Giemsa solution, dehydrated in acetone-xylene solutions, mounted in Entellan, and observed with an optical Zeiss Axioplan microscope. Images were captured with an MRc5 AxioCam digital camera and processed with the Axiovision program. Percentage of infected macrophages, and the mean number of intracellular *T. gondii* per macrophage were quantified. 200 macrophages per coverslip were counted in triplicate for each experiment.^35^

*Evaluation of NO production* - The production of NO was assessed by the Griess reagent. Supernatants were combined 1:1 with the Griess reagent for 10 min, the samples were analysed in a spectrophotometer (540 nm), and NO production in μM was obtained by comparing readings with a sodium nitrite diluted in DMEM standard curve.^37^

*Immunofluorescence of bradyzoite antigen, iNOS, phosphorylated Smad2 and LC3A* - Infected macrophages over coverslips cultured for 2, 6, 12, 24, and 48 h were collected and fixed in PBS containing 3% formaldehyde, washed and incubated for 10 min in PBS containing 0.5% Triton X-100. Cells were incubated with 100 mM ammonium chloride in PBS and incubated for 10 min in PBS containing 3% BSA (PBS-BSA). Cells were incubated for 1 h in the serum of mice chronically infected with *T. gondii* diluted 1:5000 in PBS-BSA and rabbit polyclonal antibodies that recognise BAG-1^38^ (kindly provided by Dr Louis Weiss), iNOS (M 19, Santa Cruz Biotechnology), phosphorylated Smad2 (P-SMAD2) (# 3101, Cell Signaling Technology) or LC3A (L8793, Sigma), all diluted 1:200 in PBS-bovine serum albumin (PBS-BSA). Cells were washed with PBS-BSA and incubated with secondary antibodies against mouse (Alexa 594) and rabbit (Alexa 488) antibodies, diluted 1:100 in PBS-BSA, washed with PBS, mounted in ProLong Gold containing advanced diagnostic imaging (DAPI), and observed in the Axioplan microscope equipped with an HBO100 mercury lamp. Images were captured as before and processed with Adobe Photoshop. At least 100 macrophages per coverslips were quantified discriminating nuclear positivity for P-SMAD2. At least 100 tachyzoites per coverslips associated or not to vesicle positive for LC3A were quantified. Both quantifications were performed in triplicates in three independent experiments and means and standard deviations were calculated.

*Data analyses* - Values are expressed as means and standard deviations and were analysed statistically using one-way and two-way analysis of variance (ANOVA) and Tukey-Kramer multiple comparisons test. Repetition of experiments and p values are indicated in the legends of the figures and table.

## RESULTS

**Fig. 1:**
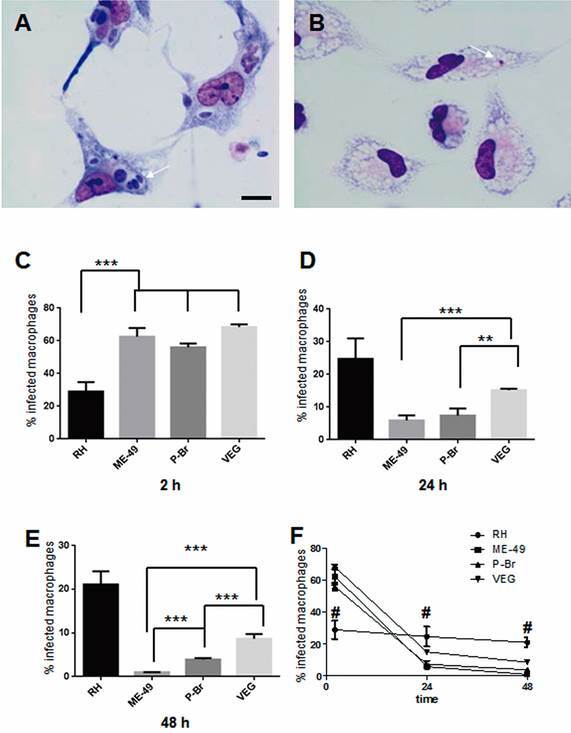
bright-field images of Giemsa stained activated mouse macrophages infected for 48 h with *Toxoplasma gondii* (arrows) of the RH (A) and ME-49 (B) strains. Bar: 5 µm. Percentage of infected macrophages after 2 (C), 24 (D), and 48 h (E) of infection of activated macrophages. Results are expressed as means ± standard deviations in triplicate of six independent experiments. A one-way analysis of variance (ANOVA) was performed, and a Tukey-Kramer multiple comparisons test was applied for comparison between strains (C, D, and E). P values are noted as: ***p < 0.001; **p < 0.01. A two-way ANOVA was performed (F) to compare the persistence of infection between RH and the other strains, # p < 0.001.

*Only the RH strain persisted in activated macrophage* - Tachyzoites of the RH strain were found with preserved morphology in activated macrophages after 24 and 48 h of infection (Fig. 1A). However, the ME-49 and P-Br strains exhibited altered morphology after 24 h and by 48 h intracellular structures resembling digested tachyzoites were observed (Fig. 1B). The VEG strain was found persisting in macrophages after 24 and 48 h (not shown), but RH was much easier to find. Quantification of the percentage of infected macrophages by *T. gondii* revealed that the RH strain presented the lowest entrance value in the macrophage population as verified after 2 h of interaction; the other three strains had similar entrance rates (Fig. 1C, F). The RH strain was able to persist in macrophages as seen by the maintenance of the percentage of infected macrophages as infection progressed (Fig. 1D-F). The persistence capacity of the VEG strain decreased after 24 and 48 h of infection but was higher compared to ME-49 and P-Br strains that almost disappeared from macrophages over the examined time points (Fig. 1D-F). The mean number of intracellular *T. gondii* for the RH strain tended to increase as infection progressed, but the other three strains decreased (not shown). Parasites of the RH strain were negative for Bag antigen after 24 h of infection (not shown), indicating their tachyzoite stage during persistence.

*All strains inhibited the NO microbicidal defense system after infection, but only the RH strain sustained this effect* - NO production was effectively inhibited by tachyzoites of the RH strain after 24 and 48 h of infection and to a lesser extent by the VEG strain (Table). ME-49 and P-Br strains were also capable of inhibiting NO production by macrophages at 24 h, but with a twofold lower capacity compared to the RH strain (Table). After 48 h macrophages infected by ME-49 strain produced more NO compared to uninfected macrophages and P-Br strain did not inhibit NO production (Table). The VEG strain also had a lower capacity to inhibit NO compared to the RH strain at 48 h (Table).

**Fig. 2:**
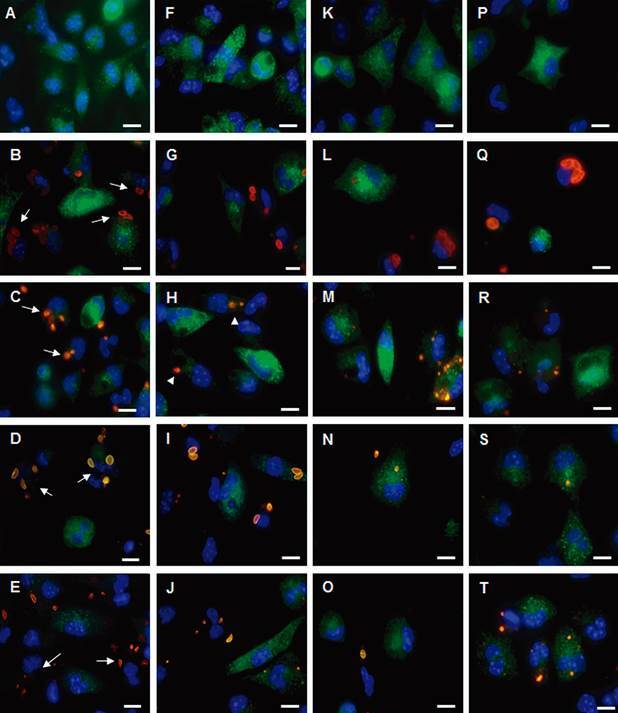
immunofluorescence of nitric oxide synthase (iNOS) expression (green) and *Toxoplasma gondii* (red) in infected macrophages (blue nuclei). Macrophages after 2 h without infection (A) or after infection with strains RH (B), ME-49 (C), P-Br (D), or VEG (E). Infected macrophages (arrow) have reduced iNOS expression. Macrophages after 6 h without infection (F) or after infection with strains RH (G), ME-49 (H), P-Br (I), or VEG (J). Macrophages infected with ME-49 (arrowhead) have an intermediate level of iNOS expression. Macrophages after 24 h without infection (K) or after infection with strains RH (L), ME-49 (M), P-Br (N), or VEG (O). Macrophages after 48 h without infection (P) or after infection with strains RH (Q), ME-49 (R), P-Br (S), or VEG (T). Bars: 10 µm. Representative images from four independent experiments.

Tachyzoites of all tested strains decreased iNOS expression by activated macrophages after 2 h of infection (Fig. 2B-E) compared to uninfected macrophages (Fig. 2A, F, K, P). The presence of tachyzoites infecting macrophages caused the low expression of iNOS with clear contrast to uninfected neighboring cells (Fig. 2B-E). After 6 h of infection, a few parasites of the ME-49 strain were observed inside macrophages that had an intermediate expression of iNOS (Fig. 2H), but P-Br and VEG strains were able to inhibit iNOS expression (Fig. 2I, J), but this inhibition was not as extensive compared to RH strain (Fig. 2G). After 24 h of infection, macrophages infected by the RH strain continue not expressing iNOS (Fig. 2L); macrophages infected with ME-49 or P-Br strains that presented *T. gondii* structures (digested tachyzoites), recovered iNOS expression (Fig. 2M, N). VEG tachyzoites maintained the inhibition capacity of iNOS expression after 24 h of infection (Fig. 2O). After 48 h, macrophages infected with the RH strain had tachyzoites with preserved morphology and inhibited iNOS expression (Fig. 2Q). In contrast, macrophages infected with ME-49 and P-Br fully recovered iNOS expression and parasite remains were difficult to observe (Fig. 2R, S); macrophages infected with VEG recovered iNOS expression (Fig. 2T).

**TABLE:** Nitric oxide production of mouse peritoneal macrophages activated with interferon-γ and lipopolysaccharide, infected with tachyzoites of the RH, ME-49, P-Br or VEG strains of *Toxoplasma gondii* ^*a*^

Macrophages	Nitrite production (µM)			
	24 h	% inhibition 24 h	48 h	% inhibition 48 h
Uninfected	31.6 ± 1.62^*b*^	-	38.3 ± 0.88	-
*T. gondii* RH	10.7 ± 1.00^*c*^	66.1	19.4 ± 0.20^*c*^	49.4
*T. gondii* ME-49	21.3 ± 0.61^*c,d,e*^	32.6	45.6 ± 1.25^*c,d,e*^	0
*T. gondii* P-Br	20.1 ± 5.75^*c,d,e*^	36.4	42.4 ± 5.20^*d,e*^	0
*T. gondii* VEG	14.7 ± 2.14^*c*^	53.5	31.2 ± 0.84^*c,d*^	18.6

*a*: macrophages were activated 24 h prior to infection with tachyzoites of the different strains of *T. gondii*. After 2 h, cells were washed, further cultured for 24 and 48 h and nitrite evaluated in the supernatant; *b*: values are means ± standard deviation of four independent experiments; *c*: significantly different (p < 0.001) from respective values for uninfected macrophages as calculated by analysis of variance (ANOVA); *d*: significantly different (p < 0.001) from respective values for RH-infected macrophages as calculated by ANOVA; *e*: significantly different (p < 0.01) from respective values for VEG-infected macrophages as calculated by ANOVA.

**Fig. 3:**
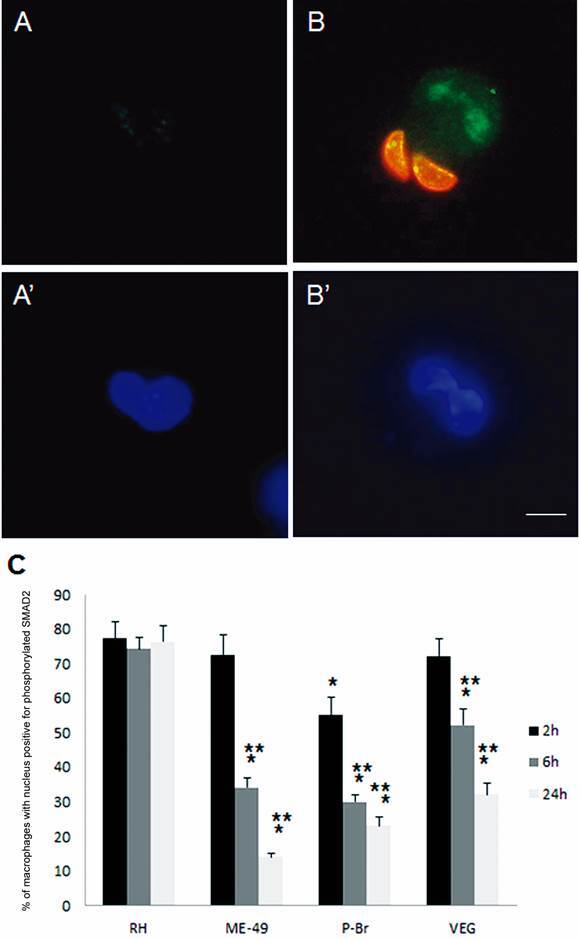
immunofluorescence of *Toxoplasma gondii* (red), nuclear translocation of phosphorylated Smad2 (green) in infected macrophages (blue nuclei). Macrophages after 2 h without infection (A, A’) or after infection with *T. gondii* (B, B’); a 2 h infection by the used strains caused similar nuclear translocation of phosphorylated Smad2. DAPI-stained nuclei of macrophages can be seen in the images with upper case letters with apostrophe (A’, B’). Bars: 10 µm. Representative images from three independent experiments. (C) Quantification of macrophages with nucleus positive for phosphorylated Smad2 in percentage after 2, 6, and 24 h of infection with the RH, ME-49, P-Br, and VEG strains. Results are expressed as means ± standard deviations in triplicate of three independent experiments. *Significantly different in relation to the value of RH after 2 h of infection by one-way analysis of variance (ANOVA) (p < 0.05). **Significantly different in relation to the value of 2 h of infection within the same strain by one-way ANOVA (p < 0.05).

*All tested strains induced TGF-β signaling, sustained only by the RH strain* - TGF-β signaling involves the phosphorylation of Smad2 that is translocated to the nucleus.^39^ This signal inhibits NO production by macrophages^40^ and is involved in the capacity of *T. gondii* to inhibit the expression of iNOS.^31^ To determine if TGF-β signaling was involved in the inhibition of iNOS expression after infection with the tested strains, nuclear translocation of P-SMAD2 during a 24 h infection period was verified. Uninfected macrophages, independent of the observed time, had a low fluorescence signal for P-SMAD2 (Fig. 3A, A’). After 2 h of infection with all tested strains, P-SMAD2 was localised in the nucleus of infected macrophages (Fig. 3B, B’); the presence and persistence of P-SMAD2 in the nucleus of macrophages were quantified along with the infection between strains (Fig. 3C). Nuclear translocation of P-SMAD2 persisted for 6 and 24 h only in macrophages infected with the RH strain (Fig. 3C). When macrophages were infected with ME-49 or P-Br strains P-SMAD2 nuclear translocation decreased as infection progressed (Fig. 3C) and infection with VEG strain caused an intermediate response in P-SMAD2 nuclear translocation between RH and ME-49 (Fig. 3C).

**Fig. 4:**
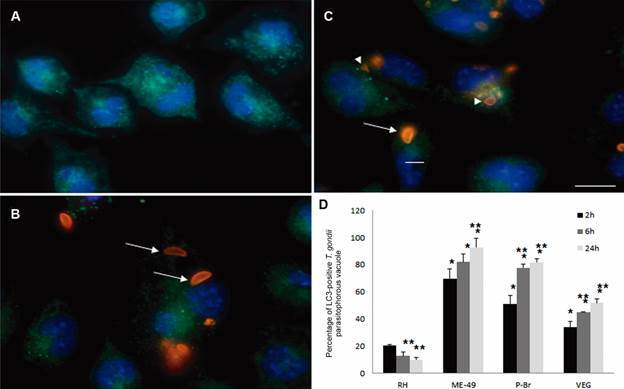
immunofluorescence of LC3 (green) and *Toxoplasma gondii* (red) in infected macrophages (blue nuclei). Activated macrophages without infection (A) or after 2 h of infection with RH (B) or ME-49 (C) strains. Tachyzoites in parasitophorous vacuoles without (arrows) or associated (arrowheads) to LC3 (green) can be observed. Bars: 10 µm. Representative images from three independent experiments. Percentage of LC3-positive *T. gondii* parasitophorous vacuoles after 2, 6, and 24 h of infection with the RH, ME-49, P-Br, and VEG strains (D). Results are expressed as means ± standard deviations in triplicate of three independent experiments. *Significantly different in relation to the value of RH after 2 h of infection by one-way analysis of variance (ANOVA) (p < 0.05). **Significantly different in relation to the value of 2 h of infection within the same strain by one-way ANOVA (p < 0.05).

*RH was the only strain that avoided LC3 recruitment to the PV* - Immunofluorescence microscopy showed that LC3 appeared as dots in the cytoplasm of uninfected activated macrophages (Fig. 4A). After 2 h of infection, LC3 was not easily associated with the PV of the different strains tested, particularly RH (Fig. 4B). However, as infection proceeded, LC3 associated more with the PV especially of the ME-49 (Fig. 4C) and P-Br strains. The association of LC3 with the PV were quantified and confirmed that the RH was the only strain of the ones used that maintained a low association of LC3 with the PV (Fig. 4D), that the ME-49 and PB-r strains had a higher association that increased with time and that the VEG strain presented an intermediate response (Fig. 4D).

## DISCUSSION

*T. gondii* has many strategies to evade the host’s immune system. One of the microbicidal systems this parasite needs to cope with is the production of NO by activated macrophages. We have demonstrated that tachyzoites of *T. gondii* of the RH strain can inhibit NO production of mice peritoneal macrophages by degrading iNOS through TGF-β signaling.^31^ To verify if this is a general evasion mechanism, tachyzoites of four strains of *T. gondii* with different virulence were used to infect activated macrophages.

RH was the only strain that could persist in activated macrophages during the infection period analysed. The persistence of tachyzoites of the RH strain in activated macrophages has been reported before,^30^ ^,^ ^31^ and as far as we know, no comparison between strains of distinct virulence has ever been reported after infection of activated macrophages. RH was the only tested strain capable of persisting in activated macrophages, VEG strain persisted less but better than ME-49 and P-Br. The RH strain was quite suitable in resisting the microbicidal mechanisms of activated macrophages.

All strains could degrade iNOS right after macrophage infection (2 h). However, recovery of iNOS expression and the subsequent NO production of infected macrophages were strain-specific. ME-49 and P-Br were the strains with the least capacity to avoid the return of iNOS expression in macrophages. Macrophages infected with VEG strain also recovered iNOS expression and NO production, but at lower levels compared to ME-49. Only the RH strain sustained this inhibition. This indicates that all strains inhibited the NO microbicidal defense system after interacting with macrophage, but RH was the only strain that sustained this effect from inside the PV. The other strains did not sustain the inhibition of iNOS expression, probably because they were killed by other microbicidal systems (discussed below) allowing the macrophage to regain the capacity to respond to IFN-γ and LPS present in the culture medium.

TGF-β signaling was assayed to further understand the evasion mechanism of *T. gondii* that inhibits the NO microbicidal defense system. Only the RH strain maintained TGF-β signaling for longer periods; the VEG strain was capable of inducing longer TGF-β signaling in comparison to ME-49 and P-Br strains that were capable of inducing it only during the initial infection. Thus, all strains were capable of inducing TGF-β signaling soon after infection, but only RH maintained this signal long enough to inhibit iNOS expression for the entire period examined. The VEG strain sustained this signaling for a longer period compared to the ME-49 and P-Br strains, explaining its improved capacity to persist in macrophages.

Finally, we examined the loading of LC3 to the PV in macrophages. PV of activated mice peritoneal macrophages harboring parasites of the RH strain presented the least LC3 loading concerning the other strains, indicating a strain-dependent response in this host cell. It has been demonstrated that LC3 loads to the PV of IFN-γ activated HeLa cells in a strain-specific manner with type I strain being able to better avoid this decoration than type II and III strains.^24^ Thus, it was not surprising that in activated macrophages LC3 loaded to the PV in a strain-specific way. LC3 loading to the PV is necessary for IRG accumulation onto the PV resulting in *T. gondii* growth arrest.^19^ ^,^ ^20^ ^,^ ^21^ IRGs control type II and III *T. gondii* strains,^12^ ^,^ ^15^ ^,^ ^23^ but type I has special ROPs (5, 17, and 18) that phosphorylate IRGs, neutralising their microbicidal effect against the parasite.^11^ ^,^ ^13^ The persistence of type I *T. gondii* strains in IFN-γ activated mice host cells indicates that these ROPs are virulence factors.^13^ Therefore, in addition to the capacity of type I strains to avoid IRGs by their unique ROPs, the capacity to impair LC3 loading to the PV adds to the evasion mechanisms of this type of strain, explaining its higher virulence in mice. This is further corroborated by the analysis of the influence of ROP 5, 17, and 18 on the capacity of *T. gondii* to inhibit NO production of activated mice macrophages.^33^ We have recently demonstrated that ROP 5, 17, and 18 are not essential for NO production inhibition after infection of activated mice macrophage. The lack of these ROPs in knockouts of the RH strain background was not essential for their persistence in activated macrophages; these mutants were not killed, sustaining macrophages in a non-activated state as seen by low NO production.^33^ Thus, these RH knockouts were probably escaping death due to the lower LC3 loading, which reduces the association of IRGs to the PV. This results in better survival and maintenance of NO production inhibition. The capacity of the RH strain to avoid LC3 loading to the PV as described here probably increases parasite survival in these macrophages, resulting in persistence, the sustainability of the TGF-β signaling, and NO production inhibition. On the other hand, ME-49 (type II), PB-r (recombinant), and VEG (type III) were capable of avoiding NO production during the initial hours of infection but probably did not sustain NO production inhibition because they could not avoid the IRG microbicidal system causing their death, return of macrophage response to IFN-γ and LPS, and re-expression of iNOS and NO production. Collectively, the capacity of the RH strain of *T. gondii* to avoid LC3 loading to the PV in activated macrophages is probably an advantage over the other three strains used in this work, leading to higher persistence in activated macrophage.

All strains examined in this work were capable of inducing TGF-β signaling that deactivates macrophages leading to iNOS degradation and inhibition of NO production. However, the virulent strain (RH), contrary to the other strains used, also could avoid LC3 loading to their PV. The evasive mechanism based on NO production inhibition may be present in *T. gondii* due to the presence of this microbicidal system in most hosts infected by this parasite. Thus, this inhibition capacity may be a general evasive strategy of all strains of *T. gondii*.
